# Machine Learning Uncovers Food- and Excipient-Drug Interactions

**DOI:** 10.1016/j.celrep.2020.02.094

**Published:** 2020-03-17

**Authors:** Daniel Reker, Yunhua Shi, Ameya R. Kirtane, Kaitlyn Hess, Grace J. Zhong, Evan Crane, Chih-Hsin Lin, Robert Langer, Giovanni Traverso

**Affiliations:** 1 David H. Koch Institute for Integrative Cancer Research, Massachusetts Institute of Technology, Cambridge, MA 02139, USA; 2 Division of Gastroenterology, Hepatology and Endoscopy, Department of Medicine, Brigham and Women’s Hospital, Harvard Medical School, Boston, MA 02115, USA; 3 MIT-IBM Watson AI Lab, Massachusetts Institute of Technology, Cambridge, MA 02139, USA; 4 Department of Mechanical Engineering, Massachusetts Institute of Technology, Cambridge, MA 02139, USA; 5 Lead Contact

## Abstract

Inactive ingredients and generally recognized as safe compounds are regarded by the US Food and Drug Administration (FDA) as benign for human consumption within specified dose ranges, but a growing body of research has revealed that many inactive ingredients might have unknown biological effects at these concentrations and might alter treatment outcomes. To speed up such discoveries, we apply state-of-the-art machine learning to delineate currently unknown biological effects of inactive ingredients—focusing on P-glycoprotein (P-gp) and uridine diphosphate-glucuronosyltransferase-2B7 (UGT2B7), two proteins that impact the pharmacokinetics of approximately 20% of FDA-approved drugs. Our platform identifies vitamin A palmitate and abietic acid as inhibitors of P-gp and UGT2B7, respectively; *in silico*, *in vitro*, *ex vivo*, and *in vivo* validations support these interactions. Our predictive framework can elucidate biological effects of commonly consumed chemical matter with implications on food-and excipient-drug interactions and functional drug formulation development.

## In Brief

Reker et al. use machine learning to identify biological activities of food and drug additives. Validation confirms vitamin A palmitate as an inhibitor of P-glycoprotein transport and abietic acid as an inhibitor of UGT2b7 metabolism. Such associations have important implications as food-or excipient-drug interactions.

## Graphical Abstract

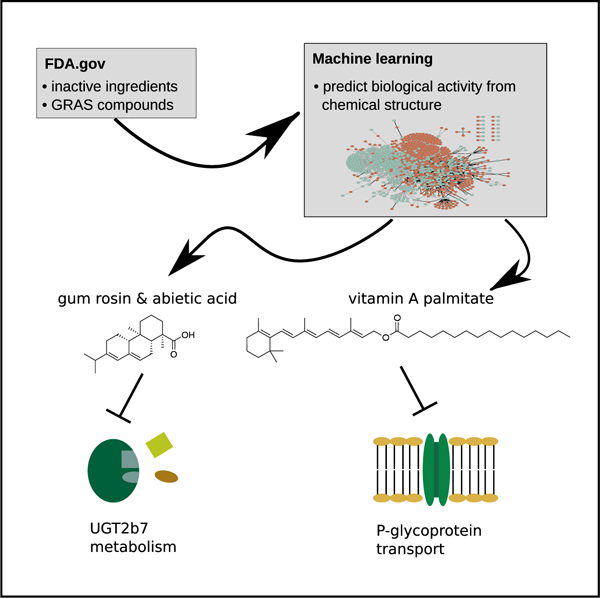

## INTRODUCTION

**Figure 1.**
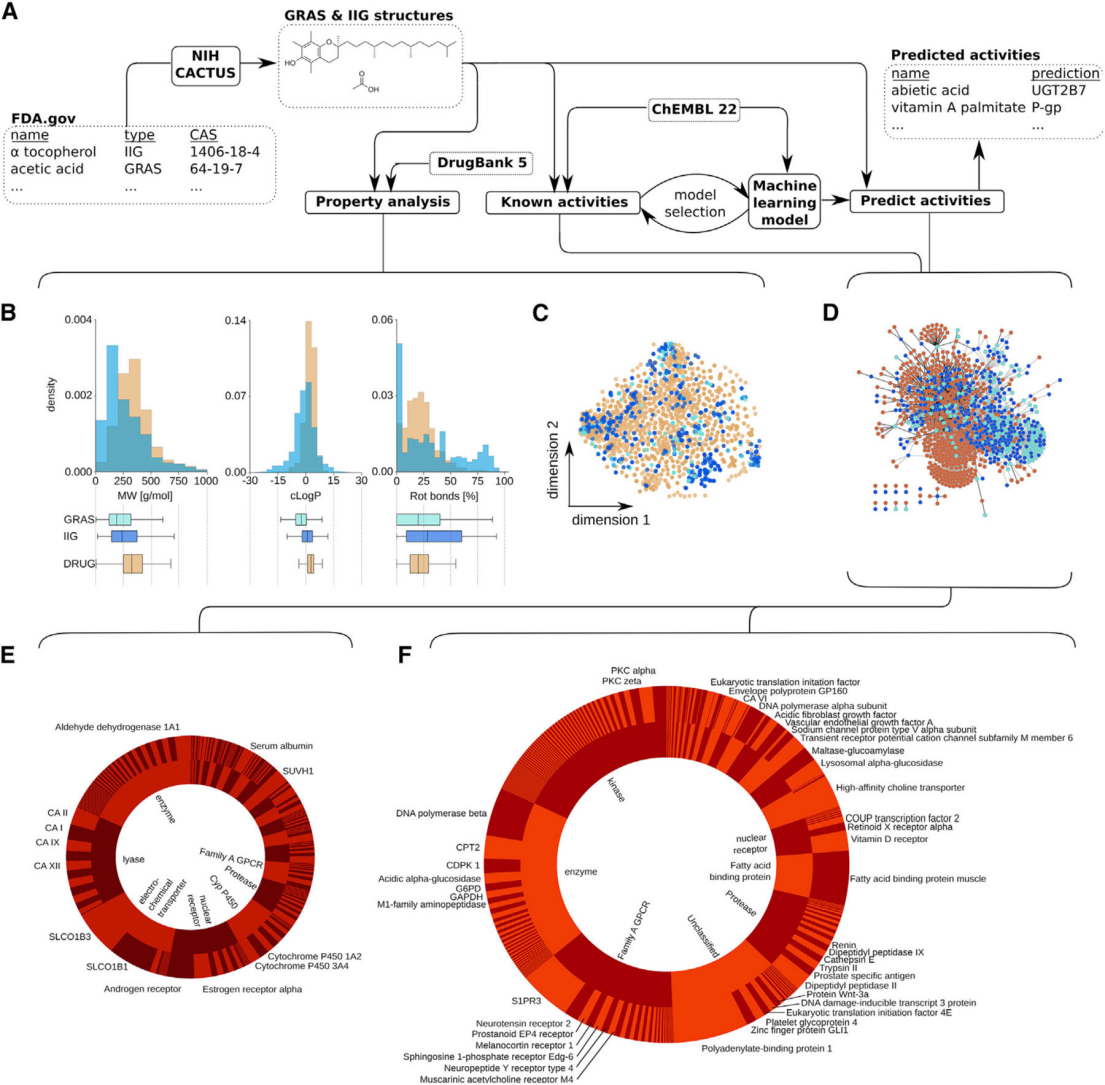
Inactive Ingredients and GRAS Compounds Resemble FDA-Approved Drugs and Exert Known or Potentially Novel Bioactivities (A) Schematic visualizing the general workflow of the study and the utilized datasets. Briefly, CAS numbers for generally recognized as safe (GRAS) and inactive ingredient (IIG) compounds were extracted and curated from the FDA website (https://www.fda.gov) and translated into SMILES structural representations using the CACTUS NIH webserver (https://cactus.nci.nih.gov). These chemical representations were then harnessed to calculate physicochemical properties (http://rdkit.org) and compare the property distributions with approved drugs (https://www.drugbank.ca). Biological activity data were extracted from ChEMBL22 (http://ebi.ac.uk/chembl) to identify previously reported activities for GRAS/IIG compounds and build machine learning models (https://scikit-learn.org) to predict additional biological activities of GRAS/IIG compounds. (B) Distribution of molecular weight (MW), calculated logP, and the fraction of rotational bonds (rot bonds) among GRAS (light blue) and IIG (dark blue) compared to FDA-approved drugs in the DrugBank database (DRUGS, orange). Summary statistics represented through boxplots show considerable overlap in the three distinct distributions. (C) Visualization of chemical space spanned by GRAS (light blue) and IIG (dark blue) compared to approved drugs stored within the DrugBank 5.0 database (orange). Projection based on t-Distributed Stochastic Neighbor Embedding (t-SNE) using Morgan fingerprints (r = 4, 2,048 bits; RDKit) is shown. (D) Pharmacology network of GRAS and IIG. Compounds are shown as light blue (GRAS) or dark blue (IIG) nodes; protein targets (ChEMBL22) are shown in red.A compound and a target are connected either when the compound has been previously measured to interact with the protein (black edge) or when machine learning models predicted that the compound is likely to interact with the protein (*Z* score > 4; gray edge). (E and F) Distribution of number of previously reported (left, E) and computationally predicted (right, F) activities on the level of different protein families (inner pie charts). Top seven families are labeled. Outer pie charts visualize the number of reported or predicted activities per protein. Proteins for which more than 10 GRAS or IIG compounds have been reported or predicted to modulate their activity have been annotated.

Generally recognized as safe (GRAS) chemicals (Burdock and Carabin, 2004) and inactive ingredients (IIGs) are compound collections curated by the US Food and Drug Administration (FDA), comprising natural and synthetic compounds that serve as additives in drug and food products. They are considered a reliable resource of safe chemical matter for drug delivery, formulation science, and food production. However, an exponentially growing body of research and clinical reports has contested their biologically inert character and suggests sensitive patients might experience adverse reactions to IIGs (Reker et al., 2019a). Similarly, examples of revoked GRAS status highlight the potential of unknown health effects revealed after initial GRAS assessment (FDA, 2015; Hallagan and Hall, 2009). Conversely, many GRAS/IIG compounds could have beneficial biological effects that might be currently underappreciated (Martinez-Mayorga et al., 2013). These could provide prime starting points for drug discovery and as functional foods (Martinez-Mayorga and Medina-Franco, 2014), given the well-understood safety, metabolism, and pharmacokinetics of such compounds (Burdock and Carabin, 2004). Furthermore, they might warrant the rational design of functional formulations, which will enable the translation of therapeutics to patients that are currently restricted through unfavorable liberation, absorption, distribution, metabolism, excretion, and toxicity (LADMET) profiles. However, such applications require the systematic identification of biological effects of GRAS/IIG compounds, which is costly and restricted by compound availability and assay throughput. We hypothesized that machine learning could provide an economical and innovative approach to identify beneficial or adverse biological effects of such compounds (Figure 1A). Harnessing the wealth of publicly available biochemical data, machine learning drastically decreases the necessary time and resources to unravel the effects of small molecules on (patho-)biologically relevant macromolecules. We and others have provided predictive models to assess the biological effects of natural products (Rodrigues et al., 2016), but it is unknown whether machine learning can provide biologically relevant predictions for the natural products within the GRAS/IIG repositories. Here, we use state-of-the-art machine learning to predict biologic targets of GRAS/IIG compounds to gain further insights into the biological effects of these essential compound classes and provide innovative starting points for drug discovery and drug formulation research.

## RESULTS

### IIGs and GRAS Compounds Resemble Drugs and Have Been Previously Measured in Biological Assays

We began our analysis with a comprehensive investigation of molecular properties and structures of a total of 799 IIG and GRAS compounds (Table S1). Interestingly, both IIG and GRAS compounds resemble approved drugs (DrugBank 5.0; Wishart et al., 2018), according to many important molecular properties (Table S2), most notably (Figure 1B) the fraction of rotatable bonds, the molecular weight, and the predicted logP (cLogP). Using two-dimensional depictions of chemical space based on descriptions of chemical substructures, we observed a substantial overlap between GRAS/IIG compounds and approved drugs (Figure 1C). These data suggest that there seems to be no underlying physicochemical or chemical (sub-)structure distinction between GRAS/IIG compounds and approved drugs, supporting the potential for GRAS/IIG to exert relevant biological effects. Indeed, many of the GRAS/IIG compounds have been previously measured in functional or phenotypic assays and can elicit relevant biological activity: a total of 877 positive assay readouts have been confirmed for GRAS and IIG compounds according to ChEMBL22 data (Figure 1D), which compiles data both from the literature as well as from larger screening efforts (Bento et al., 2014). Strikingly, we found acetaminophen (39 activities) to be the compound in our collection with the highest number of reported positive measurements. Given its role as a therapeutic and its associated liver toxicity, its inclusion in the FDA list of IIGs (version 0716 UNII 362O9ITL9D) is highly questionable, showcasing the importance of data curation and the utility of mining biological screening data for this purpose. The next three GRAS/IIG compounds most frequently reported to elucidate biological activity according to the literature (ChEMBL data) are all GRAS compounds with well-studied polypharmacological effect and include caffeine (34 activities), L-glutamic acid (26 activities), and tannic acid (23 activities). Such privileged structures (Rodrigues et al., 2016) provide ample opportunities for optimization and highlight the biochemically promiscuous character of material that is commonly perceived as biologically inert at low concentrations. The most common protein families that have been previously reported to be modulated by GRAS/IIG compounds (Figure 1E) are enzymes (160 activities), lyases (129 activities), electrochemical transporters (122 activities), and nuclear receptors (98 activities).

### Machine Learning Predicts Biological Associations of IIGs and GRAS Compounds

**Figure 2.**
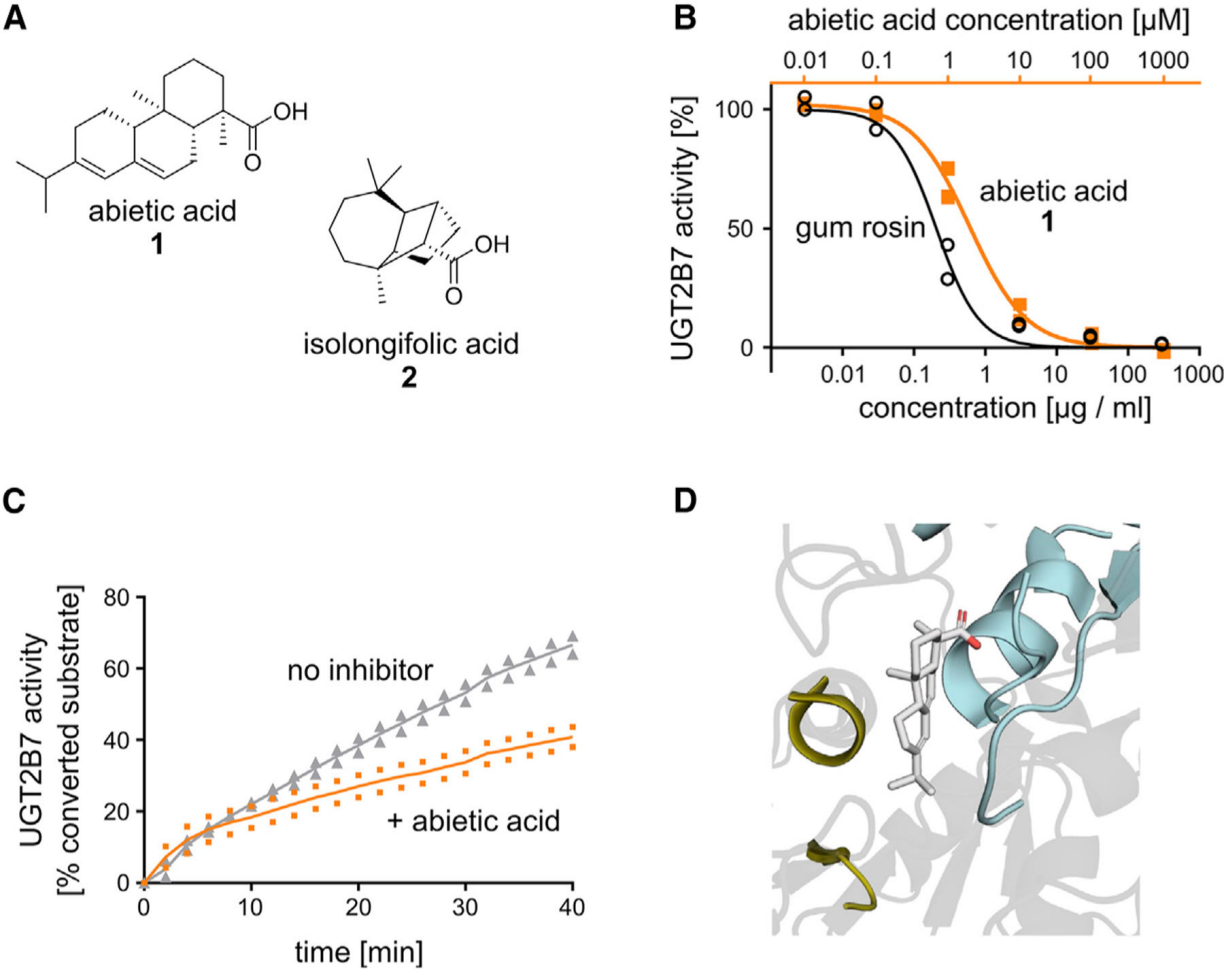
Gum Rosin and Abietic Acid Inhibit UGT2B7 Activity (A) Chemical structures of abietic acid (1) and training data compound isolongifolic acid (2). (B) *In vitro* validation shows that gum rosin (black circles) and abietic acid (orange squares) inhibit UGT2B7 activity in microsomes. (C) The effect of abietic acid (orange) on UGT activity was confirmed in complex tissue liver lysates, where it slowed the conversion of a proprietary UGT substrate (Biovision K692; gray). (D) Computational docking indicates that abietic acid has the potential to interact with UGT2B7 at the interface of the substrate- (gold) and the co-factor-binding (cyan) domains.

We harnessed these data of 877 known biological activities of GRAS/IIG compounds from ChEMBL22 data together with an additional 1,334,128 positive and negative measurements for small molecules probed for their biological activity (ChEMBL22) to construct 1,776 machine learning models to predict the modulation of protein activity (pAffinity = –log[XC_50_ or K_i/D_]) from molecular structure and physicochemical properties of GRAS/IIG molecules (Reker et al., 2016). A set of 256 known and previously reported pAffinity values of GRAS/IIG compounds against these 1,776 modeled protein targets served as a test set for model selection (Table S3). To avoid over-fitting, we excluded 21 test cases in which the Tanimoto similarity of the test compound to the training data was larger than 0.8. Our final random forest models had a mean absolute error (MAE) of 0.95 on this test set, outperforming other machine learning approaches such as support-vector machines and k-nearest neighbor models (MAE > 1.0; p < 0.001; two-tailed paired t test; cf. Table S3). This retrospective evaluation suggests that our random forest models enable us to anticipate the potency of a biological activity of a GRAS/IIG compound against the modeled protein targets, but we realized that these predicted modulations need to be contextualized on the level of the individual proteins to account for the expected activity range–which can vary widely (cf. Table S4). A mild positive correlation between molecular weight and measured affinity for most proteins in our training data (average Pearson r = 0.15; r > 0 for 78% of investigated proteins; cf. Table S4) suggested that additional normalization by molecular weight would enable us to more accurately contextualize the expected activities per protein. Notably, other properties, such as the cLogP, did not correlate with the pAffinity values (Pearson r = 0.05; cf. Table S4) in the training data and therefore were not considered for further normalization. We utilized probability proportional to size (PPS) sampling to generate a molecular weight-matched library of random chemicals (Reker et al., 2019b) to determine the expected predicted affinity for a protein target (cf. Table S4). This enabled us to interpret the predictions for GRAS/IIG structures statistically and only focus on the most promising predictions. Restricting predictions only to those whose predicted pAffinity exceeds 4 standard deviations of the mean prediction for the background dataset, we identified a total of 1,903 predicted ligand-target associations for GRAS/IIG compounds (Figure 1F)—2-fold more than currently known activities for these molecules (Figure 1D).

The three most frequently predicted targets for GRAS/IIG compounds are polyadenylate-binding protein 1 (127 predictions), fatty-acid-binding protein 3 (95 predictions), and sphingosine 1-phosphate receptor Edg-3 (89 predictions), which are implicated in oculopharyngeal muscular dystrophy (Apponi et al., 2010), cardiac fatty acid utilization (Binas et al., 1999), and multiple sclerosis (Choi et al., 2011), respectively. Overall, the three most commonly predicted protein classes are enzymes (343 predictions), kinases (343 predictions), and family A G protein-coupled receptor (280 predictions)—supporting the unmapped potential of GRAS/IIG compounds to exert adverse reactions through biological effects, act as starting points for drug discovery projects, or enhance treatments as functional supplements. Importantly, there was no strong correlation between the number of previously measured bioactivities and the number of predicted bioactivities of a GRAS/IIG compound (Pearson linear correlation r = 0.17; Figure 1C), which signifies that there is a vast uncharted polypharmacological space (Hopkins et al., 2006) of safe compounds and that our machine learning approach acts independently from previously acquired biological activity data for GRAS and IIG compounds.

### Gum Rosin and Abietic Acid Inhibit UGT2B7 *In Vitro* and *Ex Vivo*

Given the potential benefits of formulations that can improve LADMET profiles of therapeutics, we focused our investigation on GRAS/IIG compounds predicted to modulate metabolic and transport proteins. We first investigated whether machine learning would enable us to identify inhibitors of glucuronidation through UGT2B7 among IIGs. Glucuronidation is a major metabolic pathway that affects around 10% of all drugs (Williams et al., 2004). Multiple drugs and toxins have been reported as UGT2B7 inhibitors, recognized through drug-drug interactions (Bélanger et al., 2009; Williams et al., 2004), leading to significant changes in drug exposure and altering treatment efficiency and toxicity. Our machine learning model for UGT2B7 inhibition showed acceptable retrospective accuracy in 10-fold cross validation (MAE = 0.3; Table S4), encouraging us to harness this model for UGT2B7 inhibitor detection among GRAS/IIG structures. When predicting GRAS/IIG compounds with our model, we noticed a relatively narrow range of predicted activities so that we included a predictive variance threshold of 0.4 as an additional filter to focus on high-confidence predictions. Our model suggested abietic acid as one of the most promising IIGs for UGT2B7 inhibition with an estimated half-maximal inhibitory concentration (IC_50_) value of 2.8 µM. The most similar training compound with known UGT2B7 activity was isolongifolic acid (IC_50_ = 2 µM), replacing the fused ring system of abietic acid through bridged rings (Figure 2A). The computer predicted that these distinct chemical structures will lead to a similar pharmacophoric interaction pattern and provide an equivalent inhibition of UGT2B7 activity. Indeed, in our functional assay, abietic acid inhibited the activity of UGT2B7 with an IC_50_ value of 2.2 ± 0.3 µM (Figure 2B)—closely matching the computationally predicted effect. Purified abietic acid is not an FDA-approved IIG but was included in our library as one of the main ingredients of gum rosin (colophony). Gum rosin is an FDA-approved IIG and is used as a glazing agent in pills and chewing gums with E number E915. According to Pillbox data (https://pillbox.nlm.nih.gov), rosin is currently included in pills of Rifater (rifampin/isoniazid/pyrazinamide; Sanofi-Aventis US) and Chlor-Trimeton 12 Hour (chlorpheniramine maleate; Schering Plough HealthCare Products). Gum rosin’s main component abietic acid is among the most soluble and least toxic resin acids (Peng and Roberts, 2000) and is harmless in mice (Winter, 1989). We tested whether gum rosin could show the same effect in our *in vitro* assay and found an IC_50_ value of 0.21 ± 0.03 µg/mL (Figure 2B), suggesting that abietic acid with a less potent IC_50_ of 0.6 ± 0.1 µg/mL and making up about 33% of the gum rosin we obtained is a major but potentially not the only component of the multicomponent resin material (Peng and Roberts, 2000) to inhibit UGT2B7 activity (Figure 2B). To confirm these effects in a more complex biological context, we used pig liver lysate and found that abietic acid successfully inhibited UGT activity and slowed the conversion rate of UGT2B7 (Figure 2C). To study the potential binding mode of abietic acid with UGT2B7, we performed a pocket-agnostic docking study using SwissDock (Grosdidier et al., 2011) based on a homology model of UGT2B7 that was generated with SwissModel (Arnold et al., 2006). SwissDock autonomously explores multiple possible binding sites and modes and scores them according to the interaction potential of abietic acid with the amino acid residues in different target sites. The most probable binding mode identified through the software positions abietic acid at the interface between the catalytic site and the co-factor binding domain, thereby potentially disrupting the interaction of the co-factor uridine diphosphate glucuronic acid with the metabolic substrates of UGT2B7 (Figure 2D). For further contextualization of this positive result, we tested three additional IIGs that had a promising prediction albeit higher predictive variance, indicating lower predictive confidence in the estimated inhibitory potency. All three additionally tested IIGs did not modulate UGT2B activity at a testing concentration of 50 µM (Table S5), providing important additional data to further improve our understanding of the UGT2B7 structure-activity relationship (Table S1). Even more importantly, these negative readouts attest to the potential of these IIGs to be included in drug products without risking UGT2B7-mediated excipient-drug interactions—highlighting an additional use case of our platform to enable the identification of IIGs and associated pharmaceutical products with lower risk of unwanted biological effects.

### Vitamin A Palmitate Inhibits P-gp Activity

**Figure 3.**
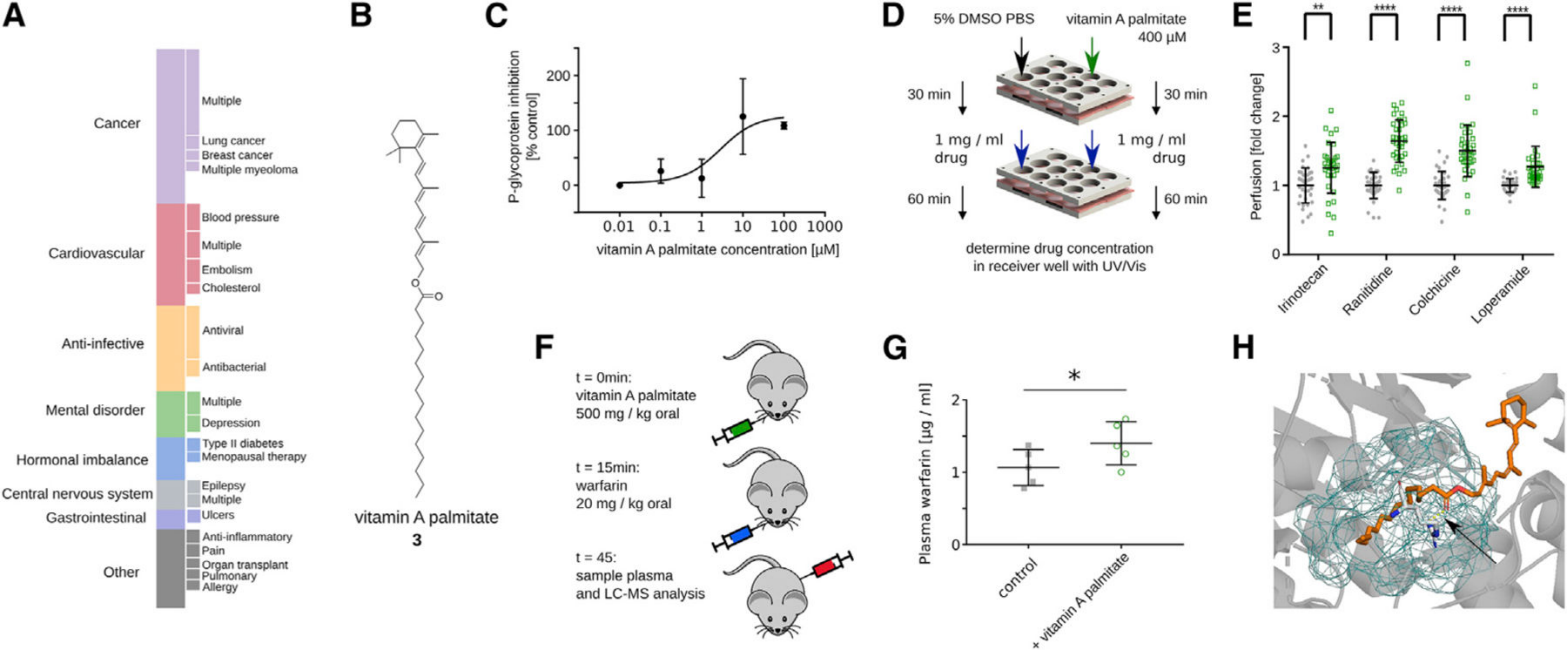
Vitamin A Palmitate Modulates P-gp Activity (A) P-gp is involved in the transport of 8% of all approved drugs, spanning a broad range of different indications (DrugBank 5.0). Complete bar corresponds to 170 approved drugs. Only sub-classifications with at least three drugs are visualized on the right. (B) Structure of vitamin A palmitate (3). (C) Vitamin A palmitate inhibits P-gp activity in HepG cells with an IC_50_ of 2.9 ± 3.6 µM. (Biovision K507) Data plotted as mean and standard deviation, curve fitted in Prism using the standard three parameter equation for “inhibitor vs. response”. (D) Schematic of *ex vivo* tissue permeability experiment in (E). (E) Vitamin A palmitate increases the permeability of the four known P-gp substrates irinotecan, ranitidine, colchicine, and loperamide across porcine intestinal tissue. p ≤ 0.001; two-tailed t test with Holm-Sidak correction. (F) Schematic of *in vivo* experiment in (G). (G) Vitamin A palmitate shows a mild increase of systemic warfarin, a known P-gp substrate, after oral delivery in mice. p = 0.04; one-tailed t test. (H) Computational docking suggests that vitamin A palmitate can bind the ATPase site of P-gp (blue mesh) with a stabilizing hydrogen bond formed with ARG1047 (dashed yellow line; see black arrow).

We next investigated whether our workflow was able to identify P-gp inhibitors among GRAS compounds. P-gp is one of the main active drug transporters, and modulation of its activity can drastically impact the pharmacokinetics of 8% of currently approved therapeutics spanning various important disease areas (Figure 3A; Sparreboom et al., 1997). Many of the top-predicted GRAS/IIG compounds, such as tannic acid (Kitagawa et al., 2007), cholesterol (Wang et al., 2000), stearic acid (Callaghan et al., 1993), vitamin E (Tang et al., 2013), beta carotene (Teng et al., 2016), and glyceryl palmitate (Konishi et al., 2004), were previously reported in the literature to modulate P-gp activity. This is encouraging because this validates our predictions given that these associations were not part of the training data. In addition to these cases, the model showed a MAE of 0.45 in retrospective 10-fold cross validations, which further increased our confidence in our model’s predictive capabilities. One of the highest scoring and previously unknown predictions of P-gp inhibition was made for vitamin A palmitate (Figure 3B), an important nutrient that is a GRAS-approved direct food ingredient. The model anticipated that vitamin A palmitate would inhibit P-gp with an estimated IC_50_ value of 5 µM. We confirmed this prediction in a cell-based *in vitro* assay, where vitamin A palmitate inhibited P-gp-mediated efflux of a fluorescent reporter substrate with an IC_50_ of 2.9 ± 3.6 µM (Figure 3C). In a high-throughput *ex vivo* Franz diffusion cell assay, vitamin A palmitate significantly increased the permeability of four FDA-approved drugs that are known P-gp substrates (Figures 3D and 3E). Further, we observed that vitamin A palmitate increases the oral absorption of warfarin in mice by ca. 31% (Figures 3F and 3G). We again used pocket-agnostic docking using the SwissDock server (Grosdidier et al., 2011) to determine the possible site of interaction for vitamin A palmitate with P-gp and found this effect might be caused by the palmitate tail occupying the ATPase site, stabilized by an additional hydrogen bond involving the P-gp arginine residue at position 1,047 (Figure 3H). Overall, the transport modulation by vitamin A palmitate could constitute an important food-drug interaction or be harnessed in formulation development for drugs with transport liabilities.

## DISCUSSION

In summary, we show that state-of-the-art machine learning based on publicly available biochemical data can be effectively harnessed to discover pharmacologically relevant targets of GRAS and IIG compounds rapidly. This further showcases the potential applications of fast and easily deployable data science tools for predicting effects of natural products in complex biological systems. It is important to keep in mind that such algorithms will heavily rely on availability of high-quality data, indicating that the identification of biomacromolecular targets of GRAS/IIGs through such pipelines will be inherently limited to proteins with known small molecular modulators. Augmenting such pipelines with advanced, high-throughput assay technology and prediction algorithms focusing on target protein structure or phenotypical readouts might further increase the scope and predictive capabilities of such workflows. Furthermore, *in silico* and *in vitro* data alone provide insufficient evidence for clinical relevance of biological activities of GRAS/ IIGs. We have here included a series of *ex vivo* and *in vivo* validations to provide additional biological context, but additional validations, such as clinical data analysis, will further increase our confidence in the relevance of such associations. Notwithstanding, the biological activities of GRAS/IIGs is an overlooked and clinically relevant research field (Reker et al., 2019a), and smart algorithms will have the potential to drive and accelerate such discoveries for personalized treatment design and drug formulation development.

## STAR★METHODS

### LEAD CONTACT AND MATERIALS AVAILABILITY

Further information and requests for resources and reagents should be directed to and will be fulfilled or coordinated by the Lead Contact, Giovanni Traverso (cgt20@mit.edu). For the distribution of materials and data, all raw data and code to make predictions is available on GitHub (https://github.com/DanReker/CellRep2020).

### EXPERIMENTAL MODEL AND SUBJECT DETAILS

All animal procedures were conducted in accordance with protocols approved by the Massachusetts Institute of Technology Committee on Animal Care. For the *in vivo* warfarin uptake experiment, female BALB/c mice between 10–12 weeks were used in this study. Animals were maintained in a conventional barrier facility with a climate-controlled environment on a 12-h light/12-h dark cycle, fed *ad libitum* with regular rodent chow. For the *in vitro* cell experiments, HepG2 cells were cultured in DMEM + 10% FBS + 1% pen-strep and kept in 5% CO2 atmosphere at 37°C.

### METHOD DETAILS

#### Datasets curation

IIG (https://www.accessdata.fda.gov/scripts/cder/iig/) and GRAS structures (https://www.accessdata.fda.gov/scripts/fdcc/?set=SCOGS) were retrieved from the FDA website (accessed June 2016) as CAS codes. The codes were converted into SMILES structures using the NIH CACTUS server (https://cactus.nci.nih.gov/cgi-bin/lookup/search) and subsequently manually curated. The curation was done in an inclusive fashion, retaining structural approximations for complex mixtures or polymeric structures, which were subsequently filtered out for prospective applications. The DrugBank database (version 5.0) was extracted in XML format and post-processed in Python to extract all SMILES strings for small molecules in the category “approved”. ChEMBL22 served as the reference database for bioactive compounds to enable machine learning-based predictions. ChEMBL22 was pre-processed in accordance with previously published protocols by Schneider and colleagues for ChEMBL data curation (Reker et al., 2016; Reutlinger et al., 2014). We focused on modeling only direct protein targets (confidence score > 6) with at least 50 unique activity annotations (IC_50_, K_i_, EC_50_). Activities were logarithmized into pAffinity values to enable model fitting over a wide-range of activities. Entries with pAffinity less than 3 or greater than 12 were excluded. Inactive compounds were annotated with a pAffinity value of 3. When matching K_i_ values with XC_50_ of the same compound measured against the same protein target, pK_i_ values are, on average, 0.41 larger than the measured XC_50_. Therefore, all pK_i_ were shifted by 0.41 to enable the mixing of K_i_ and XC_50_ data (Kalliokoski et al., 2013). To increase our dataset but capture lower activities for measurements annotated as lower bounds (“>“), we penalized these measurements by one logarithmic unit before further processing. Only activities were kept that were not labeled as inconclusive (“Insoluble,” “Not Tested,” “Not evaluated,” “Unstable,” “Not Determined”). In case multiple measurements have been reported for the same compound against the same target, we averaged multiple activity entries to create a single training data point as long as their standard-deviation was below one, otherwise this data point was labeled as inconclusive and excluded.

#### Machine learning predictions

Structures of IIG and GRAS compounds, as well as known bioactive compounds from ChEMBL22, were encoded using Morgan fingerprints (radius 4, 2048 bits) as well as physicochemical properties using the RDkit (http://rdkit.org/) in Python (version 2.7.6). These descriptors were used to build Random Forest (RF, n_trees = 500 trees, max_features = None), k nearest neighbor (kNN, k = 5, weights = ‘uniform’, distance = Euclidean), and Support-Vector regression (SVR with radial basis function kernel, degree = 3) models in scikit-learn. Model selection was performed by evaluating the mean absolute error (MAE) on the validation test set and selecting the RF model given the lowest MAE. The model was further evaluated retrospectively using ten-fold cross validation with shuffling for every investigated protein to ensure sufficient performance for the individual bioactivity models. For large-scale prioritization of predictions, we normalized the predicted pAffinity of the GRAS or IIG compounds based on the average expected pAffinity prediction of a random set of compounds extracted from ChemDB that we had subsampled to approximate the molecular weight distribution of the GRAS/IIG compound libraries through Probability Proportional to Size (PPS) Sampling. This generated standardized prediction z-scores that we used to rank computational predictions. For prospective examples, predictions were additionally prioritized according to novelty and potential exposure to the investigated ingredients while accepting lower z-scores.

#### Property comparison and polypharmacology network

For dimensionality reduction, we used t-distributed Stochastic Neighbor Embedding (t-SNE) on Morgan fingerprints (r = 4, 2048 bits) for 1000 iterations with an angle of 0.5, early exaggeration of 4.0, random initialization, a learning rate of 1000.0, using the Barnes-Hut approximations and Euclidean distances. For the polypharmacology graph, we extracted all GRAS/IIG compounds from ChEMBL22 according to chemical structure matching. We included annotations for compounds with undefined stereochemistry. Annotations that were labeled as “inactive” or “inconclusive” were excluded. All other annotations were considered “active” irrespective of the value of the measured potency. This led to a set of 877 known bioactivities for GRAS/IIG compounds. This set was further augmented by adding all 1903 predictions for GRAS/IIG compounds with a z-score of at least 4 to build the network using the Python GraphTool library. For this, we generated an edge list that connected a GRAS/IIG node with a protein target node if there was a previous association reported in ChEMBL or if our machine learning algorithm predicted an association. For visualization, the edges were positioned using the ARF spring-block layout algorithm with an opposing force of 5 and an attracting force of 10.

#### UGT2B7 inhibition assay

UGT2B7 inhibition was measured utilizing Corning® Supersomes Human UGT2B7. The inhibition of UGT2B7 was measured using the commercially-available Biovision UGT activity screening kit as previously described. (Biovsion K692) Briefly, 0.1mg/ml microsomes were mixed with alamethicin for pore-formation and a proprietary UGT ligand that loses fluorescence after glucuronidation (Biovision). Plates were incubated for 5 minutes at RT and protected from light before the enzymatic reaction was initiated through the addition of UDPGA. Loss of fluorescence was measured after 30 minutes on a microplate reader (Infinite M200, Tecan) and compared to the loss of fluorescence in the presence of different concentrations of gum rosin or abietic acid dissolved in PBS with 1% DMSO. Diclofenac (1mM in PBS 1% DMSO) served as positive inhibitor control.

#### UGT tissue assay

Compound mixtures were prepared at 500 µM. The porcine liver tissue was placed in ice-cold UW solution (Bridge to Life Solutions LLC, Columbia, SC). A 4 mm biopsy punch was used to obtain liver tissue samples, followed by homogenization using a tissue homogenizer (Bertin Precellys). The sample was separated using centrifugation and the supernatant was extracted as a test sample. Two independent experiments with two different liver extracts were performed as described for the microsomes.

#### P-gp inhibition assay

HepG2 cells were used as model cells with MDR1 expression. Cells were plated at 40,000 cells per well in 200 µl DMEM + 10% FBS + 1% pen-strep. Cells were incubated overnight in 5% CO_2_ atmosphere at 37°C. Cells were then washed with PBS. Subsequently, cells were incubated with different concentrations of vitamin A palmitate in 1% DMSO PBS or 100 µM verapamil as the positive control. A proprietary, fluorogenic P-gp substrate (Biovision K507) was added and the sample was protected from light and incubated at 37°C in a 5% CO_2_ atmosphere. Fluorescence of the substrate (excitation 488 nm, emission 532 nm) was measured after 12h.

#### P-gp tissue assay

Fresh porcine intestinal tissue was washed according to previously published protocols. Briefly, porcine small intestine was procured from a local abattoir and washed exhaustively with cold PBS until the solution was clear. A high-throughput screening system as described previously was setup as described previously. Briefly, the reservoir plate was sealed with a transparent seal and each well of the reservoir plate was filled with PBS. The tissue was placed on top of the reservoir plate with the luminal side facing up, and fixed using the sample plate via magnetic force. Each well was treated with 50 µL of a 400 µM vitamin A palmitate solution in PBS with 5% DMSO or buffer control (5% DMSO in PBS) and incubated at room temperature for 30 minutes. After the incubation period, the pre-treatment was washed off completely with PBS and subsequently the sample wells were re-filled with 50 µL of one of the test drug solutions. For these solutions, one of four P-gp substrates (Irinotecan, Ranitidine, Colchicine, or Loperamide; all purchased from Sigma Aldrich) were prepared in a 5% DMSO PBS solution at concentrations of 1 mg/mL. After 60 minutes, permeability was assessed by comparing drug concentration in the receiver wells of the vitamin A palmitate treatment to the buffer control. Irinotecan was detected using UV-VIS fluorescence (excitation 370, emission 470), and Ranitidine, Colchicine and Loperamide were detected using absorption at 312 nm, 350 nm, and 415 nm, respectively.

#### P-gp *in vivo* experiment

A suspension of 500 mg/kg vitamin A palmitate in 10% DMSO PBS or 10% DMSO PBS buffer control were administered orally to five female BALB/c mice 15 minutes prior to treatment. Mice were then treated orally with warfarin 20 mg/kg. Blood was sampled after 30 minutes of oral Warfarin administration. All experiments were approved by the MIT Committee on Animal Care.

Warfarin serum concentrations were determined using Ultra-Performance Liquid Chromatography-Tandem Mass Spectrometry (UPLC-MS/MS). Analysis was performed on a Waters ACQUITY UPLC®-I-Class System aligned with a Waters Xevo® TQ-S mass spectrometer (Waters Corporation, Milford MA). Liquid chromatographic separation was performed on an Acquity UPLC® BEH C18 (50mm × 2.1mm, 1.7 µm particle size) column at 50°C. The mobile phase consisted of aqueous 0.1% formic acid, 10mM ammonium formate solution (Mobile Phase A) and acetonitrile: 10 mM ammonium formate, 0.1% formic acid solution (95:5 v/v) (Mobile Phase B). The mobile phase had a continuous flow rate of 0.6 mL/min using a time and solvent gradient composition. For the analysis of warfarin, the initial composition, 100% Mobile Phase A, was held for 1.00 minutes, following which the composition was changed linearly to 20% Mobile Phase A over the next 0.25 minutes. The composition was then changed to 0% Mobile Phase A at 2.50 minutes. The composition of 0% Mobile Phase A and 100% Mobile Phase B was held constant until 3.00 minutes. The composition returned to 100% Mobile Phase A at 3.25 minutes and was held at this composition until completion of the run, ending at 4.00 minutes, where it remained for column equilibration. The total run time was 4.00 minutes. The mass to charge transitions (m/z) used to quantitate warfarin and internal standard etoroxib were 309.07 > 163.05 and 359.02 > 279.86 respectively. Sample introduction and ionization was by electrospray ionization (ESI) in the positive ionization mode. Waters MassLynx 4.1 software was used for data acquisition and analysis. Stock solutions of warfarin and etoroxib were prepared in methanol at a concentration of 500 µg/mL. A twelve-point calibration curve was prepared in analyte-free, blank serum ranging from 1.25–5000 ng/mL. 40 µl of each serum sample was spiked with 80 µl of 250 ng/mL etoroxib in acetonitrile to elicit protein precipitation. Samples were vortexed, sonicated for 10 -minutes, and centrifuged for 10 minutes at 13,000 rpm. 100 µl of supernatant was pipetted into a 96-well plate containing 100 µl of water. Finally, 2.50 µL was injected onto the UPLC-ESI-MS system for analysis.

#### Abietic acid quantification in gum rosin

Abietic acid and gum rosin stock solutions were dissolved in methanol at a concentration of 1 mg/ml. Standard dilutions were prepared in a range of 2.5–500 µg/ml in acetonitrile. Gum rosin samples were prepared at 500 µg/ml in acetonitrile. Abietic acid was measured by High-Performance Liquid Chromatography (HPLC) on an Agilent 1260 Infinity II HPLC system (Agilent Technologies, Inc.) equipped with a Model 1260 quaternary pump, Model 1260 High Performance autosampler, Model 1260 thermostat, Model 1260 Infinity Thermostatted Column Compartment control module, and Model 1260 diode array detector. Data processing and analysis was performed using OpenLab CDS ChemStation (Agilent Technologies, Inc.). All solvents used were purchased from Sigma-Aldrich Corporation. Chromatographic separation was carried out on an Agilent Poroshell 120 EC-C18 4.6×50mm, 2,7 µm analytical column maintained at 55°C. The optimized mobile phase consisted of isocratic 0.1% aqueous formic acid (1%) and acetonitrile (99%) at a flow rate of 1.50 ml/min over a 4 min run time. The injection volume was 10 µl, and the selected ultraviolet (UV) detection wavelength was 242 nm.

#### Computational docking

The crystal structure of human P-glycoprotein was extracted from the PDB (PDB: 6c0v) and the cytosolic portion without any bound ATP was isolated in PyMol. UCSF Chimera was used for pre-processing of the structure using “dock prep” with default parameters. The molecular structure of vitamin A palmitate was extracted from PubChem and transformed into a MOL2 file in KNIME. Docking was performed on the SwissDock server. The top scoring binding mode with an estimated ΔG of –8.71 kcal / mol was extracted using UCSF Chimera and visualized in PyMol. For visualizing the ATPase domain, a mesh was created from atoms surrounding the co-cry-talized ATP with a maximal distance of 5 Å.

A homology model of human UGT2B7 was created using the SwissModel server based on the amino acid sequence of UGT2B7 as stored in Uniprot (UniProt: P16662). The top-scoring homology model was based on a crystal structure for UGT85H2 (PDB ID 2pq6.1) and was used for docking in SwissDock. The molecular structure of abietic acid was provided via its ZINC ID (ZINC2267806). The highest scored binding mode with an estimated ΔG of –7.79 kcal / mol was extracted using UCSF Chimera and visualized in PyMol. For visualization, residues corresponding to the catalytic domain (AA33–37 and AA149–153) as well as the co-factor binding domain (AA356–398) were colored gold or cyan, respectively.

### QUANTIFICATION AND STATISTICAL ANALYSIS

Pearson correlation coefficients were calculated in Python to determine relationships between different variables. To compare differences in mean values of distributions, we calculated two-sample, two-sided t tests in Python. When comparing the performance of the different machine learning models on the GRAS/IIG test data, paired two-sided t test statistics were calculated in KNIME. For all *in vitro*, *ex vivo*, and *in vivo* experiments we used n ≥ 2. Exact sample sizes and *p* values are reported in the figure captions and at the corresponding positions of the main manuscript. Significant changes were defined as p < 0.05. *p* values were represented in plots as follows: p > 0.05, ‘n.s.’ (not significant, may not be indicated); p ≤ 0.05, ‘*’; p ≤ 0.01, ‘**’; p ≤ 0.001, ‘***’; p ≤ 0.0001, ‘****’. Plots were generated in matplotlib using Python. For boxplots, the line shows the median, the box outlines the lower and upper quartile values (Q1 and Q3, 25% and 75% of the data). The whiskers extend to the highest and lowest datum that is not considered an outlier, where the outlier threshold is defined by default as 150% the interquartile range (IQR) from Q1 or Q3.

## Supplementary Material

1

2

3

4

5

6

## Figures and Tables

**Table:** KEY RESOURCES TABLE

REAGENT or RESOURCE	SOURCE	IDENTIFIER
Biological Samples		

Freshly extracted porcine liver	Massachusetts Institute of Technology -Division of Comparative Medicine	N/A
Freshly extracted porcine intestine	Massachusetts Institute of Technology -Division of Comparative Medicine	N/A

Chemicals, Peptides, and Recombinant Proteins		

vitamin A palmitate (> 1700000 USP units per g)	Sigma Aldrich	R1512
Gum rosin (Oleoresin from various species of Pinus, Portugal)	Sigma Aldrich	60895
Abietic acid (> 90%)	VWR	AA42582-MD
Irinotecan hydrochloride (> 97%)	Sigma Aldrich	I1406
Ranitidine hydrochloride (> 98%)	Sigma Aldrich	R101
Colchicine (> 95%)	Sigma Aldrich	C3915
Loperamide hydrochloride (> 98%)	Sigma Aldrich	L4762
Warfarin (> 98%)	Sigma Aldrich	A2250
Ursodiol (99.6%)	Sigma Aldrich	PHR1579
Alpha-terpinol (≥98.5%)	Sigma Aldrich	04899
Menthol (> 98.5%)	Sigma Aldrich	M2772
Supersomes Human UGT2B7	Corning	456427

Critical Commercial Assays		

UGT activity assay	Biovision	K692
MDR1 Ligand Screening Kit	Biovision	K507

Deposited Data		

FDA Inactive Ingredients	https://www.accessdata.fda.gov/scripts/cder/iig	0716 UNII 362O9ITL9D
FDA Generally Recognized As Safe	https://www.accessdata.fda.gov/scripts/fdcc/?set=SCOGS	June 2016
DrugBank	https://www.drugbank.ca/	5.0
UniProt – sequence of human UGT2B7	https://www.uniprot.org	P16662
PDB – structure of human P-gp	http://www.rcsb.org	6c0v
ChEMBL database of bioactivities	https://www.ebi.ac.uk/chembl/	22

Experimental Models: Cell Lines		

Human: HepG2	ATCC	HB-8065


Experimental Models: Organisms/Strains		

Mouse: BALB/c	Charles River	028

Software and Algorithms		

Python	https://www.python.org	2.7
RDKit	http://rdkit.org	201309–1
Scikit-learn	https://scikit-learn.org	0.14.1
SwissModel	https://swissmodel.expasy.org	N/A
SwissDock	http://swissdock.ch	N/A
GraphTool	https://graph-tool.skewed.de	2.18
PyMol	https://pymol.org	2.2.0
UCSF Chimera	http://cgl.ucsf.edu/chimera/	1.13.1
Data and prediction code	https://github.com/DanReker/CellRep2020	N/A

## Highlights

- Machine learning predicts biological effects of excipients and GRAS compounds
- Abietic acid and gum rosin inhibit UGT2b7 metabolism *ex vivo*
- Vitamin A palmitate inhibits P-glycoprotein transport *in vivo*
- Such associations can cause unknown drug interactions
