# Supplementary material for: Machine Learning Uncovers Food- and Excipient-Drug Interactions
Source: Cell Rep. Author manuscript; Available in PMC 2020 Apr 23. (PMC7179333; doi:10.1016/j.celrep.2020.02.094)
Supplement: 1 [file NIHMS1578467-supplement-1.pdf]

Cell Reports, Volume *30*

Supplemental Information

Machine Learning Uncovers Food- and Excipient-Drug Interactions

Daniel Reker, Yunhua Shi, Ameya R. Kirtane, Kaitlyn Hess, Grace J. Zhong, Evan Crane, Chih-Hsin Lin, Robert Langer, and Giovanni Traverso

Supplementary Table 5. Related to Figure 2. Measured UGT2B7 activity for three additional GRAS/IIG compounds at 50 μM testing concentration.

**Compound**	**% UGT2B7 activity**
Ursodiol	91 ± 2%
Alpha-terpineol	92 ± 3%
Menthol	94 ± 4%
